# Still Far to Go With Characterisation of Molecular and Genetic Features of Diffuse Large B-Cell Lymphoma in People Living With HIV: A Scoping Review

**DOI:** 10.3389/or.2024.1375291

**Published:** 2024-04-19

**Authors:** Maudy C. P. Manyau, Blessing Zambuko, Moses Chatambudza, Danai T. Zhou, Justen Manasa

**Affiliations:** ^1^ Laboratory Diagnostic and Investigative Sciences, University of Zimbabwe, Harare, Zimbabwe; ^2^ Biomedical Research and Training Institute, Harare, Zimbabwe; ^3^ Lancet Laboratories, Pathology, Harare, Zimbabwe

**Keywords:** non-Hodgkin lymphoma, HIV/AIDS, molecular pathology histogenesis, CD10, BCL6, cyclin H, MUM1, CD138

## Abstract

Diffuse large B-cell lymphoma (DLBCL) accounts for half of non-Hodgkin lymphoma cases in people living with human immunodeficiency syndrome (PLWH). The interplay of viremia, immune dysregulation and co-infection with oncogenic viruses play a role in pathogenesis of DLBCL in PLWH (HIV-DLBCL). This scoping review aimed to describe the molecular landscape of HIV-DLBCL, investigate the impact of biomarker on clinical outcomes and describe technologies used to characterise HIV-DLBCL. Thirty-two papers published between 2001 and 2023 were included in this review. Samples of HIV-DLBCL were relatively small (16–110). Cohort effects influenced frequencies of molecular characteristics hence their impact on survival was not clear. Molecular features were distinct from HIV-unrelated DLBCL. The most frequently assessed characteristic was cell of origin (81.3% of studies). Somatic mutations were the least researched (6.3% of studies). Overall, biomarker identification in HIV-DLBCL requires broader richer data from larger or pooled samples using more powerful techniques such as next-generation sequencing.

## Introduction

Despite the introduction of combination antiretroviral therapy (cART), people living with HIV (PLWH) remain at a 9–11 fold higher risk of developing non-Hodgkin lymphoma (NHL) when compared to the general population [1–3]. Globally, diffuse large B-cell lymphoma (DLBCL) is the most frequent subtype, which accounts for 30%–50% of NHL cases in PLWH [4, 5]. There is considerable variability in the clinical course of DLBCL. Some of this variability has been explained by the International Prognostic Index (IPI). Regarding PLWH, immune status and viral load further refine risk stratification [6].

The clinical heterogeneity in DLBCL represents underlying differences in the molecular characteristics of the malignancies [7]. This heterogeneity has been explained in part by the cell of origin (COO) classification. Specifically, germinal centre B-cell like (GCB) DLBCL, activated B-cell like (ABC) DLBCL and a heterogenous and unclassifiable group [7, 8]. The COO classification has been confirmed using immunohistochemistry (IHC) based algorithms [9]. However, IHC does not always agree with GEP. Additionally, the utility of IHC-based algorithms in predicting survival is questionable in the rituximab era [10]. This scoping review aims to synthesize evidence for the applicability of COO determined GEP and IHC predicting survival in PLWH.

Next-generation sequencing (NGS) has further delineated DLBCL into distinct prognostic groups [11]. Genetic subtypes have also provided insight into pathogenesis of DLBCL. For instance, B-cell receptor (BCR) signalling via activity of constitutive nuclear-factor kappa-beta (NF-κβ), have been implicated in the development of ABC-DLBCL [11–13]. GCB-DLBCL is dominated by alterations in epigenetic modifiers, G-protein migration pathway proteins, indirect modifiers of BCRs and PI3K signalling [14–16]. BCL6 structural variants and NOTCH2 gene mutations have been reported to be present in subsets of ABC, GCB and unclassified DLBCL [11, 13]. Classification algorithms such as Lymphgen^®^ rely on these seed mutations and other co-occurring genetic aberrations to classify DLBCL [17].

While advances have been made in the elucidation of pathogenic mechanisms, classification, and prognostication of DLBCL, it is not clear whether these apply to PLWH. Pathogenesis of NHL in PLWH is complex, and there is evidence to support the role of impaired immune surveillance, immune dysregulation, oncogenic viruses and antigen stimulation [18–20]. The present scoping review aimed to describe the distribution of molecular and genetic characteristics of DLBCL in PLWH, investigate their impact on survival, and assess the extent that newer technologies are being used to characterize HIV-DLBCL.

## Materials and Methods

This was performed according to PRISMA guidance for scoping reviews [21].

### Search Strategy

MEDLINE (Pubmed), Embase and Google Scholar databases were searched for literature published between January 2000 and March 2023. The electronic search was performed by combining Medical Subject Headings (MeSH) or Embase equivalent using the following search terms: *HIV “*acquired *immune deficiency syndrome*,*”* “*diffuse large B-cell lymphoma”* OR *“large B-cell lymphoma*,*” pathology*, and *survival.* The list was filtered for age ≥18 years, and human studies published between 2000 and 2023. A supplementary search was conducted using Google Scholar. The results were sorted according to relevance, and only the first 200 results were screened as per general guidance [22]. Bibliographic references of the included articles were examined for additional citations.

### Inclusion and Exclusion Criteria

Studies included were:

• Original research publications
• PLWH with comorbid DLBCL (±HIV-uninfected individuals)
• Those that included molecular and genetic characterization data

Studies excluded were:

• Reviews, meta-analyses, case reports and commentaries
• Studies which only included patients with one type of DLBCL

### Data Extraction

Eligible articles were assessed by two researchers (PM and BZ). In the event of disagreements, a third researcher was consulted, and disagreements were resolved by consensus. Publications were first screened by title, then abstract and finally full text. Data extracted from each study included: study characteristics, COO and biomarkers distribution, analytical methods and prognosis if available. Data were entered into standardized tools. Where the patient population included other NHL sub-types and/or HIV-uninfected cases numeric data were reported for HIV-DLBCL cases, otherwise only a narrative description was reported.

## Results and Discussion

**FIGURE 1:**
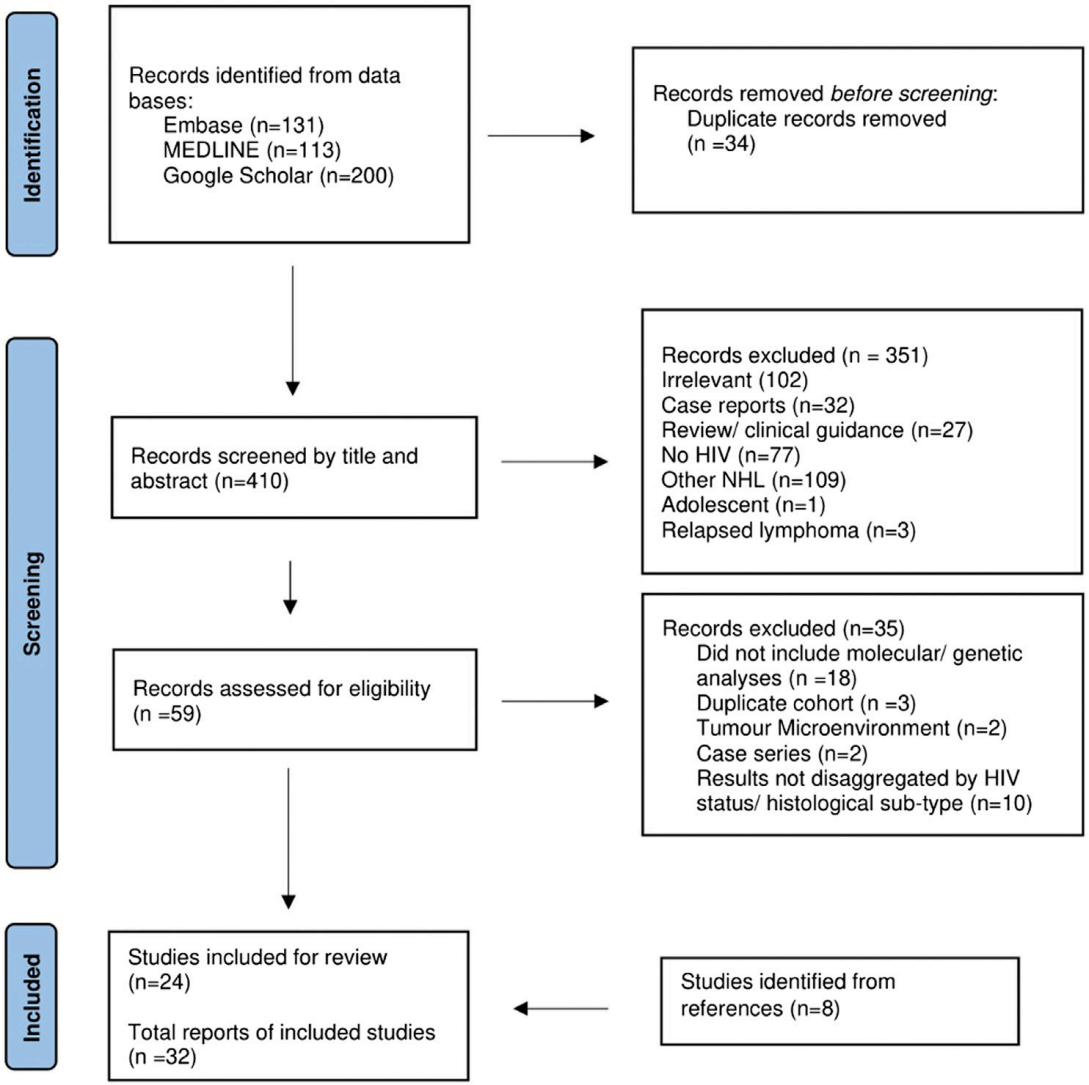
Flow diagram of studies included from Embase, MEDLINE and Google Scholar searches.

Details of the records retrieved, and study selection are provided in Figure 1. A total of 32 publications from 2001 to 2023 were retrieved. The publication types included 26 original articles, 4 conference abstracts and 2 correspondences (Table 1).

**TABLE 1:** Study characteristics.

Authors and publication year	Patient population and sample size	Molecular/genetic features assessed	Analytical methods	COO algorithm	Outcomes
Baptista, 2022 [23]	74 HIV-NHL (47 DLBCL, 24, BL, 3 HGBL)	Histogenesis, EBER1 rearrangements (BCL2, BCL6, MYC)	IHC, FISH, ISH	Hans	5-year OS and PFS
Baptista, 2019 [24]	47 HIV-NHL (42 DLBCL, HGBL-DH, HGBL NOS)	Histogenesis, EBER1, Lymph2Cx panel	IHC, GEP	Hans and LLMPP code set	5-year OS and PFS
Besson, 2017 [25]	110 HIV-NHL (52 DLBCL, 23 BL, 14 plasmablastic, 21 other)	Histogenesis, EBER1	IHC, ISH	Hans	2-year OS and PFS
Capello, 2009 [26]	43 HIV-NHL (28 DLBCL) and 105 IC-NHL	250,000 SNPs, CNVs, methylation patterns	Microarrays, Methylation-specific PCR, RT-PCR	ND	ND
Capello, 2011 [27]	116 immunodeficiency NHL (46 HIV-DLBCL, 7 HIV-PEL, 7 HIV-PCNSL, 2 PBL, 28 HIV-BL, 22 PTLD, 2 MTX-DLBCL, 2 CVID-DLBC)	Histogenesis, EBER1, CD79A/B, EZH2	Exon sequencing, IHC, ISH	Hans	ND
Carbone 2001 [28]	87 HIV-NHL (16 DLBCL, 19 BL, 22 PCNSL, 11 PEL, 12 IBL) 16 HIV-HL	Histogenesis, LMP1, EBER1	IHC, ISH	Model developed by authors	ND
Cassim, 2020 [29]	181 DLBCL NOS (50 HIV-DLBCL, 131 IC-DLBCL)	Histogenesis, EBER, Ki67, c-MYC, BCL2	IHC, ISH	Hans	5-year OS
Chadburn, 2009 [30]	81 HIV-DLBCL	Ki67, FOXP, BLIMP-1, BCL2, EBV, Histogenesis	IHC	Hans	Median OS and EFS, 1-year OS and EFS
Chao, 2012 [31]	70 HIV-DLBCL	Histogenesis, EBER1, cyclin D2, cyclin E, cMYC, p27, SKP2, (BCL6, FOXP1, PKC-beta 2, CD21, CD10, BCL2, p53, survivin, BAX, GAL3, and BLIMP1, MUM1, Ki-67, CD44, CD30, CD43, LMO2, MMP9	IHC, ISH	Hans	2-year mortality
Chao, 2018 [32]	80 HIV-DLBCL	Same as Chao, 2012	IHC, ISH	Hans	2-year mortality
Chapman, 2021 [33]	30 HIV-DLBCL	Histogenesis -BCL2, MYC, BCL6 rearrangements, 334 gene panel	GEP, FISH, WES	LLMPP code set	1-year OS
Deffenbacher, 2010 [34]	24 HIV-NHL (16 DLBCL, 2 HD, 1 FL, 1 histiocytic, 4 HGBL-NOS)	Histogenesis, EBNA1, WWOX, CNVs	Q-PCR, RT-PCR, GEP array CGH	Wright LPS	ND
Dunleavy, 2009 [35]	45 CD20^+^ HIV-NHL (35 DLBCL, 10 BL)	Histogenesis, EBER1	IHC	Hans	Median OS and PFS
Fedoriw, 2020 [36]	59 DLBCL (32 HIV-DLBCL, 27 IC-DLBCL)	CD3, CD30, CD45, CD138, BCL2, c-MYC, Ki67, HHV8, Histogenesis	IHC, ISH, whole transcriptome sequencing	Hans, Wright LPS	Median survival
Fedoriw, 2020b [37]	21 HIV-DLBCL	Human endogenous retroviruses	Computational assessment of whole transcriptome sequence data (HervQuant algorithm)	NA	OS
Hoffmann, 2015 [38]	89 HIV-NHL (58 DLBCL, 27 BL/BLL, 1 plasmacytoma, 3 unclassified)	Histogenesis, CD3, CD10, CD20, CD38, CD138), BCL-6, MUM1/IRF4, BCL-2, LMP1, Ki67	IHC, ISH	Chang model	Median OS, DFS
Kwee, 2011 [39]	204 DLBCL (19 HIV-DLBCL, 36 PT-DLBCL, 149 IC-DLBCL)	Minimal common regions, EBV	Microarrays	ND	Median OS
Little, 2003 [40]	39 HIV-NHL (31 DLBCL, 7 BL, 1 PEL)	Histogenesis, CD20, BCL2, Ki67, EBER1	IHC, ISH	ND	Median OS, PFS
Madan, 2006 [41]	39 DLBCL (12 HIV-DLBCL, 27 IC-DLBCL)	Histogenesis, CD10, BCL6, Cyclin H, MUM1, CD138, PAK1, CD44, BCL2, Ki67, CD3, CD20, EBC, cMYC translocation	cDNA microarray, IHC, ISH, FISH	Model developed by authors	ND
Maguire, 2019 [42]	66 DLBCL (27 HIV-DLBCL, 36 IC-DLBCL)	Ki67, BCL2, CNVs, PanCancer pathways panel	IHC, array CGH, GEP	LLMPP code set	ND
Morton, 2014 [43]	167 DLBCL (51 HIV-DLBCL, 116 IC-DLBCL)	Histogenesis, BCL6, CD10, GCET1, LMO2, CL2, FOXP1, CD138, MUM1, TP53, EBER1, rearrangements (MYC, BCL2, BCL6)	IHC, ISH, FISH	Tally	Median OS
Ota, 2014 [44]	207 HIV-lymphomas (104 HIV-DLBCL, 58 BL, 17 PBL, 8 PEL, 8 HL, 2 MCD, 10 other)	Histogenesis, CD3, CD10, CD20, CD30, CD38, CD45RO, CD79a, CD138, BCL-2, BCL-6, IRF4/MUM1, cIgM, immunogloblin light- chain lambda, kappa, Ki67 (MIB-1), LMP-1, EBNA-2, EBER, LANA-1, MYC rearrangements	IHC, ISH, FISH	Chang model	1-year OS
Pather and Patel, 2022 [45]	115 DLBCL (93 HIV-DLBCL, 22 IC-DLBCL)	Histogenesis, BCL2, CD20, Ki67, cMYC	IHC, Dual-colour chromogenic ISH	Hans	Median OS
Petrowski, 2015 [46]	291 DLBCL (51 HIV-DLBCL, 240 IC-DLBCL)	Histogenesis	IHC	Hans	5-year OS
Phillips, 2015 [47]	NR	Micro-RNA 21	NR	NR	NR
Philippe, 2019 [48]	52 HIV-DLBCL	Histogenesis, BCL2, LMP1, MYC, Ki67, EBER-1	IHC, ISH, FISH	Hans	Median OS and PFS
Ramos, 2020 [49]	86 HIV-NHL (61 DLBCL, 15 PBL, 7 PEL, BCL, BL)	Histogenesis, BCL2, c-MYC, EBER	IHC, ISH, FISH, qPCR	Hans	CR, 12-, 24-, 36-month OS and EFS
Rusconi, 2018 [50]	66 HIV-DLBCL	Histogenesis, BCL2, c-MYC, Lymph2Cx panel	IHC, GEP	Hans and LLMPP code set	2-year OS and PFS
Shponka, 2020 [51]	130 DLBCL (72-HIV DLBCL)	AID, CD40, DC-SIGN, BCL2, MYC, COO Lymph2Cx	IHC, GEP	Hans and LLMPP code set	ND
Wang, 2023 [52]	273 HIV-DLBCL	NR	NR	NR	2-year OS
Xicoy, 2010 [53]	98 DLBCL (34 HIV-infected, 64 HIV-uninfected)	CD10, BCL6, MUM1, CD138/syn-1, BCL2	IHC	Hans	5-year OS
Vaghefi, 2006 [54]	28 NHLs (12 BL, 10 DLBCL, four PCNSL, 2 TCLs)	CNVs, EBER1/2, LMP1, EBVA2, CD20	FISH, IHC, CGH	ND	ND

Abbreviations: BCL–B‐cell lymphoma, BL, Burkitt lymphoma; BLL, Burkitt‐like lymphoma; cART, combination antiretroviral therapy; CGH, comparative genomic hybridization; CNV, copy number variation; DH, double hit; DLBCL, diffuse large B‐cell lymphoma, EBNA‐EBV, nuclear antigen, EBER ‐ EBV‐encoded RNA, EBV, Epstein‐Barr virus; FISH, fluorescent *in‐situ* hybridization; GEP, gene expression profiling; GWAS, genome‐wide association study; HGBL, high‐grade B‐cell lymphoma, HL, Hodgkin’s lymphoma, IC‐NHL, immunocompetent non‐Hodgkin’s lymphoma, IHC, immunohistochemistry; LLMPP, Lymphoma/Leukemia molecular profiling project, LANA, latency‐associated nuclear antigen, LMP1 ‐ latent membrane protein‐1, LPS, linear prediction score; MCD, multicentric Castleman’s disease, MTX‐DLBCL, methotrexate‐induced diffuse large B‐cell lymphoma, ND, not determined; NOS, not otherwise specified; NR, not reported; PCNSL, primary central nervous system lymphoma; PEL, primary effusion lymphoma; PTLD, post‐transplant lymphoproliferative disease, Q‐PCR, quantitative PCR, RT‐PCR, real‐time polymerase chain reaction; SNP, single nucleotide polymorphism, SSH‐ suppression subtractive hybridisation, TCL–T‐cell lymphoma.

### Histogenesis of HIV-DLBCL

**FIGURE 2:**
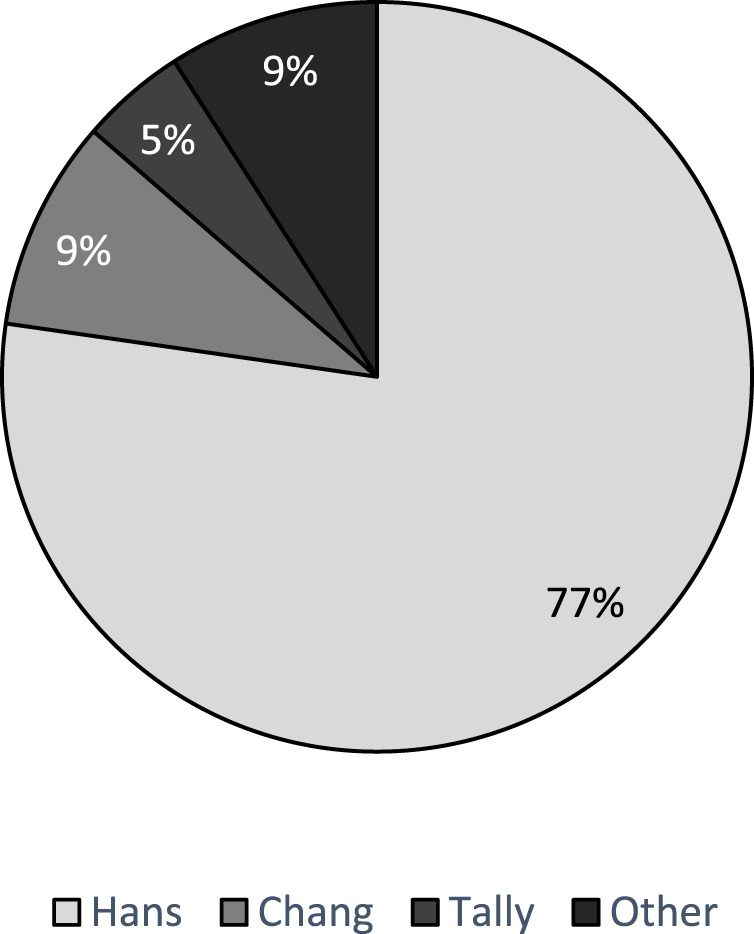
Distribution of IHC-based algorithms used to determine COO.

Histogenesis was determined in 81.3% (26/32) of the studies. Of these, GEP was used in 27.0% of studies (7/26). COO was determined using the Wright Linear Prediction score (LPS) in two studies [34, 36], and the Lymphoma/Leukemia molecular profiling project (LLMPP) code set was used in the remainder (5/7) [24, 33, 42, 50, 51]. Four studies used a combination of GEP and IHC-based algorithms to assign COO [24, 37, 50, 51]. The distribution of IHC algorithms used in 22 studies is presented below as Figure 2, and the Hans algorithm as the most frequent. Two studies developed novel histogenic models [28, 41].

Chadburn et al found that co-expression of GC and NGC markers was more frequent in HIV-DLBCL. Co-expression of CD10, BCL6 and MUM1 was observed in 19% and 6% for HIV-infected and -uninfected, respectively [30]. This potentially impacts distributions observed for COO, and subsequent prognostic assessments. Two studies assessed agreement of IHC classification methods with GEP [24, 50]. Using an inter-rater reliability test, Rusconi et al obtained a Cohen’s Kappa score of 0.6 (*p* < 0.001) when testing for Hans algorithm vs. GEP, and the authors concluded that the methods were in agreement [50]. Conversely, disagreement between the two methods was observed using McNemar’s test in the study by Baptista et al [24]. These findings suggest a need for validation of IHC-based algorithms in PLWH.

The distributions of COO varied widely between studies. Five studies presented the distribution of COO as determined by GEP [24, 33, 36, 42, 50]. The distribution of GCB ranged from 48% to 70%, ABC ranged from 18% to 48% and 4% to 25% were unclassified [24, 33, 36, 42, 50]. According to 19 studies presenting frequencies of COO subtypes using validated IHC algorithms, the GC-DLBCL frequencies ranged from 17% to 72% [23, 24, 26, 29–33, 35, 36, 43–46, 48–50, 53, 55]. The wide variability in COO between studies may have been due to cohort effects. Philippe et al stated that pooling of pre- and post-cART samples results in heterogeneity which contributes to conflicting results for impact of COO or other biomarkers [48]. In the current review, wide variability in COO has been observed. Studies which included pre-cART (recruitment/accrual before 1995) cases tended to have higher frequencies of NGC (62%–83%) [43, 44, 53] when compared to cART era cohorts (28%–61%) [25, 29, 30, 32, 35, 37, 45, 46, 48–50, 55].

Of the 12 studies which assessed the impact of COO on survival, only two found statistically significant association with survival. Dunleavy et al. found that GC-DLBCL was associated with better OS and PFS. On multivariate analysis, the NGC subtype was associated with a HR of 14.5 [35]. One-year survival for a Japanese cohort were 82% and 43% for GC and NGC-DBLCB respectively (*p* = 0.01 without censoring) [44]. However, baseline characteristics were not balanced between the two subtypes. Risk factors were more frequent in NGC-DLBCL. These included lower CD4^+^ cell counts, EBV-positivity and CNS involvement [44]. Therefore it is unclear if the association of COO with survival would still be observed on multivariate analysis. NGC-DLBCL was also associated with immunosuppression, poor performance status, advanced stage and EBV-infection in three other studies [24, 43, 46]. Patient level pooled analyses which adjusts for potential confounders may assist in teasing out the prognostic impact of COO.

### Tumour Markers

All tumour markers were determined using lymphoma biopsies. Table 2 shows the frequency of tumour markers which was characterised by wide variability. This may be attributed in part to varying positivity thresholds and cohort effects.

**TABLE 2:** Marker overexpression and prognosis.

Pathway	Marker	Frequency	Prognosis/(citation)
Cell cycle promoters	MYC	14%–58%	↔ [23, 32, 45, 50]
			↑ [49]
B-cell activators/differentiation	BCL6	28%–87%	↔ [32]
	FOXP1	37%–62%	↔ [30, 32]
	CD10	20%–53%	↔ [32]
			↓ [38]
	CD138/syn1	0%–16%	↔ [53]
	MUM1	14%–75%	↔ [32, 53]
	Blimp1	10%–28%	↔ [30, 32]
Apoptotic regulators	BCL2	16%–60%	↔ [30, 32, 40]
			↑ [48, 50]
	p53	12%–64%	↔ [40]
			↓ [32]
Other	CD20	74%–99%	↔ [32, 53]
			↓ [38]
	Ki67	16%–85%	↔ [32, 48]
			↑ [36]
			↓ [30]
	DPE	10%–42%	↑ [23, 36]
			↔
	LMO2	50–55	↔ [32]

Key: ↔ No change, ↑ increased risk, ↓ reduced risk.

DPE, double protein expression (MYC ± BCL2/BCL6).

The impact of tumour marker expression on survival was generally conflicting. Out of the five studies which assessed the impact of MYC positivity, only Ramos et al found an association between MYC and poorer survival [23, 32, 45, 49, 50]. In the study by Ramos et al MYC-positivity was associated with lower event free survival (EFS) (*p* = 0.019), when data were stratified by COO, significance remained for GC-DLBCL only [49]. Baptista et al reported a statistically insignificant trend towards increased risk of 5-year disease recurrence in MYC-positive cases (*p* = 0.055) [23]. BCL2-positivity was associated with poorer survival in two of five studies as highlighted in Table 2, one of which an adjusted HR of 4.48 (*p* = 0.05) [48, 50]. MYC/BCL2 co-expression was more frequent in NGC-DLBCL [23], and was associated with poorer survival in two of three studies where prognosis was assessed [23, 36].

Tumour proliferation marker Ki67 expression is generally elevated in HIV-DLBCL vs. immunocompetent-DLBCL (IC-DLBCL), as reported by four out of five studies [29, 36, 42, 45]. Regarding survival, there were conflicting findings for Ki67-positivity and prognosis. Chadburn and Fedoriw reported approximately a 3-fold increase in risk for mortality at the end of the follow-up period [30, 36]. Chadburn et al analysed AIDS Malignancy Consortium (AMC) trial data, and suggested that impact of Ki67 may be treatment based. Pooled data from AMC 010 and 034 trials, showed that cases with Ki67 ≥ 90% tended towards superior survival (log-rank *p* = 0.02). When stratified by trial, the trend was maintained for AMC034 only (*p* = 0.05). AMC034 participants received etoposide containing regimens and AMC010 did not [30]. Lastly, two other studies did not find an association between Ki67 and survival [32, 48]. This indicates that statistical tests for prognosis are influenced by differences in study populations, and future analyses should consider treatment effects.

HIV-infection has been shown to induce expression of activation induced cytidine deaminase (AID), an enzyme required for somatic hypermutation known to facilitate mutation of non-immunoglobulin genes such as MYC. Shponka et al investigated AID expression in PLWH and HIV-uninfected DLBCL cases. They found that AID was more frequent in PLWH, suggesting a potential pathogenic pathway in a subset of HIV-DLBCL [50].

Most of the studies included in this scoping review conducted univariate analysis of tumour markers and survival. Chao et al developed a multivariate model which considered over 40 relevant clinicopathological variables. Markers which best predicted survival were CD44, IgM and p53.

### Cytogenetics

In total nine studies described chromosomal aberrations. Five studies compared recurrent minimal common regions (MCRs) between HIV-DLBCL with IC-DLBCL using CGH based assays [26, 34, 39, 42, 54]. Three of these studies went on to compare genomic profiles between HIV-NHL subtypes with IC-DLBCL [26, 39, 42], whilst two other studies compared profiles between two different HIV-NHL subtypes [34, 54]. Chromosomal rearrangements of MYC, BCL6 and BCL2 were assessed five studies [23, 33, 43, 45, 49]. Frequency of chromosomal rearrangements are provided in Table 3. Pather and Patel also assessed copy number changes of MYC, and 57% of cases overexpressing the MYC had increased in gene copy number [45].

**TABLE 3:** Frequency of BCL2, BCL6, or MYC translocation.

Rearrangement	Frequency (%) range	Citation
MYC	12–56	[23, 33, 43, 45, 49]
BCL6	5–38	
BCL2	0–4	[23, 33, 43, 45]
Double Hit	4–7	[31, 46]

With regard to the relationship between of COO and rearrangements, MYC rearrangements may be more frequent in GC-DLBCL [23, 43] and BCL2/BCL6 rearrangements were almost exclusive to NGC-DLBCL [33, 43]. BCL6 rearrangement appears to be exclusive to EBV-negative DLBCL [33]. No other clinicopathological features were reported to be associated with MYC, BCL2 and BCL6 rearrangements, in the five studies which investigated chromosomal rearrangements.

Prognosis of rearrangements analysed in three of the five studies, and none of the rearrangements were associated with survival [23, 45, 49]. Baptista et al found a trend towards poorer survival in cases with a MYC translocation (*p* = 0.06) [23]. Generally, sample sizes were small and analyses were limited. Better powered studies with more extensive analyses are required for further evaluation of prognostic importance BCL2, BCL6 and MYC rearrangements.

Two studies which assessed the impact of HIV-infection status on genomic complexity had conflicting results [26, 42]. Capello et al reported that CNCs between HIV-infected and -uninfected cases were similar [26]. However, Maguire et al found that both amplifications and deletions were more frequent in HIV-uninfected cases [42]. Notwithstanding, both studies concluded that the pattern of CNCs were differed by HIV-status.

Regions which were consistently altered in HIV-DLBCL include 18q, 3p14.3 and 16q23.1 [26, 34, 39, 42]. 18q contains loci for BCL2 and NFATC1, and gains were exclusive to IC-DLBCL and losses characterised HIV-DLBCL [26, 39, 42]. 3p14.3 contains fragile site FRA3B and FHIT, losses in this region were present in approximately 25% of HIV-DLBCL [26, 34, 39]. This loss was accompanied by losses in 16q23.1 which contains fragile site FRA16D and involves the WWOX gene [26, 34]. Deletions in 3p14.3 and 16q23.1 corresponded to lower expression of tumour suppressor genes FHIT and WWOX, and were implicated lymphomagenesis [26]. Conflicting findings were obtained in a smaller study which analysed 4 cases where 16q deletions were not associated with WWOX gene expression levels [34]. However, the 4 cases may not have been representative of 16q deletions in HIV-DLBCL. Losses in the fragile sites were more common in ABC lymphomas [34]. Capello et al also noted that while IC-DLBCL was characterised by deletions affecting the whole chromosome, HIV-DLBCL deletions were short interstitial deletions confined within fragile site associated gene [26].

Only one study reported prognosis of recurrent MCRs [39]. Deletions in 18q, 3p and gains in 2p were associated with poor OS in HIV-DLBCL cases. Prognostic significance of 18q losses was confined to HIV-DLBCL and not IC-DLBCL [39]. Gains in 1q are found in 18%–21% of HIV-DLBCL cases [26], however, these do not appear to be prognostic [39].

### Single Gene/Pathway Somatic Mutations

Only two studies provided data on single gene/pathway somatic mutations [27, 33]. Chapman et al, performed an assessment of a custom 334 gene panel on 30 HIV-DLBCL cases. The most frequently mutated genes were TP53 (37%), MYC (30%), STAT3 (27%), HIST1H1E (23%), EP300 (20%), TET2 (20%), SOCS1 (17%) AND SGK1 (17%). When the genetic classification algorithm LymphGen was applied only 50% of cases were classifiable as follows: EZB (5/30), ST2 (4/30), A53 (3/30), BN2 (2/30) and MCD (1/30). No cases fell into the N1 class [33].

GC-DLBCL are enriched for EZH2 mutations, however, EZH2 mutation frequency is lower than expected in HIV-DLBCL (4.2%–10%) [27, 33]. Further, NGC-DLBCL are expected to be enriched for CD79A/B mutations, but these were v absent in 46 HIV-DLBCL cases [27]. The low prevalence and/or absence of these key mutations in HIV-DLBCL are likely the reason why up to 50% of cases could not be classified by the LymphGen algorithm [33]. These findings suggest different pathogenesis of HIV-DLBCL and may necessitate HIV-specific classification methods.

### Transcriptomic Profiles

Gene expression was assessed in four studies. One of these investigated expression of human endogenous retroviruses (HERVs) [37]. The other three compared GEP between HIV-infected and -uninfected cohorts [34, 36, 42].

Using the computational tool hervQuant, approximately 2,500 HERVs were identified and 19% of these were associated with OS in a dose-dependent manner. HERV expression was dependent on cART duration and not CD4 cell counts [37]. These data provide some insights on impact of HERV expression on prognosis. However, it is not clear whether they play a role in lymphomagenesis. Further, clarity is needed on whether HERV expression is a surrogate for immune status or aggressiveness of HIV-DLBCL.

The remaining three studies assessed differential gene expression between HIV-DLBCL and IC-DLBCL. Gene set enrichment analysis (GSEA) was performed using various Human Molecular Signatures Database (MSigDB) collections. Overall, pathways involved in cell cycle and DNA damage response are upregulated in HIV-DLBCL and B-cell receptor signalling pathways do not appear to have a prominent role in lymphomagenesis of HIV-DLBCL. Further, HIV-DLBCL is enriched for the extrinsic apoptotic (FAS) pathway vs. the intrinsic pathway [34, 42]. Differentially regulated pathways between HIV-infected and -uninfected DLBCL cases are presented in Table 4.

**TABLE 4:** Pathway regulation relative of HIV-DLBCL relative to IC-DLBCL.

Gene set	Gene regulation relative to HIV-uninfected	Citation
Cell Cycle	Up	[34, 42]
DNA Repair	Up	
Franconi	Up	
ATR-BRCA	Up	
Mismatch repair	Up	
E2F targets	Up	
B-cell receptor signalling	Down	
PI3K events in ERBB2 signalling	Down	
MAPK signalling	Down	
Intrinsic pathway for apoptosis	Down	
MYC targets	Up	
FAS pathway	Up	[34]
mTOR	Down	[34]
ARF pathway	Up	[34]
Hypoxia	Up	[37]

Fedoriw et al applied an unsupervised principle component analysis (PCA) on RNA-sequence data from a Malawian DLBCL cohort (HIV-infected and -uninfected). PCA revealed 2 clusters. One of the clusters contained 82% of the HIV-DLBCL cases and it was enriched for the following pathways: hypoxia, myogenesis, metabolism, angiogenesis, NOTCH and epithelial mesenchymal transition. However, when pathway regulation was stratified by HIV-infection status, only hypoxia remained significant [36].

### Epstein-Barr Virus

Sixteen studies assessed EBV infection status. The most commonly used method was EBER1-ISH. Other methods included LMP1 and EBNA1. Only one study assessed viral infection pattern. The frequency of EBV-infection varied widely from 15% to 78% [28–31, 33–36, 38, 39, 43, 44, 48, 49, 54]. Five of the fifteen studies included cases from before 1996 (pre-cART era) [34, 38, 43, 44, 54], and four of these reported EBV-infection ≥60% as detected by EBER-ISH [34, 43, 44, 54]. Hoffman et al included pre-cART cases, and using LMP1 expression EBV-infection was 20% [38]. Differences may have been due to the capacity of the different methods to detect all the latency patterns of EBV.

Where assessed, EBV infection was consistently associated with low baseline CD4 cell counts [31, 33, 35, 44]. EBV-infection was more frequent in NGC-DLBCL [30, 31, 33, 44, 54], however statistical significance was only reached in two studies [43, 44]. Other tumour markers which were associated with EBER1-positivity included BLIMP1 and CD30 [31]. Two studies reported more mutations in EBV-negative HIV-DLBCL [26, 33]. Genetic mutations which are associated with EBV included STAT3, TP53, EP300, SKG1, and histone modifying genes [33]. EBV infection was also reported to alter gene expression, where EBV-positive tumours had increased expression of IL4R, IL2RB, IL12, Ras and p53 pathways. IL5 and mTOR pathways were downregulated [34].

Nine of the 16 studies investigated prognosis of EBV-infection [23, 29–31, 33, 35, 38, 44, 48]. EBV-infection was associated with poorer survival in only three of these studies [31, 35, 44]. Overall it appears that EBV-infection does not affect survival, however it does alter molecular characteristics of HIV-DLBCL.

### Study Limitations

Eighty-four percent (27/32) of the retrieved studies were retrospective, which may have introduced limitations regarding confounding variables. Due to the descriptive nature of scoping reviews and widely conflicting findings we were not able to determine the impact of tumour markers on survival. The wide variability observed for frequencies of molecular characteristics as well as conflicting findings can be better addressed by pooled analyses.

## Conclusion

COO and tumors marker expression using IHC are the most researched characteristics of DLBCL. These were significantly impacted by cohort effects and it is still not clear if the typically accepted tumour markers are prognostic in HIV-DLBCL. Few studies investigated genomic and transcriptomic characteristics of HIV-DLBCL. However, the presented data indicates that the genomic landscape of HIV-DLBCL differs from IC-DLBCL. Dysregulation of immune pathways due to HIV-infection disrupts normal cytokine and chemokine pathways leading to replicative stress as shown by upregulation of DDR genes. Further, EBV-positive HIV-DLBCL presents as an entity with a unique genetic signature. Understanding these intricate interactions and incorporating GEP and gene sequencing will enhance diagnostic precision and population specific biomarker identification.
